# Occurrence of Bacterial and Protozoan Pathogens in Red Foxes (*Vulpes vulpes*) in Central Italy

**DOI:** 10.3390/ani12202891

**Published:** 2022-10-21

**Authors:** Valentina Virginia Ebani, Chiara Trebino, Lisa Guardone, Fabrizio Bertelloni, Giulia Cagnoli, Simona Nardoni, Emily Sel, Emily Wilde, Alessandro Poli, Francesca Mancianti

**Affiliations:** 1 Department of Veterinary Science, University of Pisa, Viale delle Piagge 2, 56124 Pisa, Italy; 2 Centre for Climate Change Impact, University of Pisa, Viale delle Borghetto 80, 56124 Pisa, Italy; 3 Struttura Semplice Section of Genoa-Portualità, Istituto Zooprofilattico Sperimentale del Piemonte, Liguria e Valle d’Aosta, 16129 Genoa, Italy

**Keywords:** *Vulpes vulpes*, red fox, bacterial pathogens, protozoan pathogens, zoonoses

## Abstract

**Simple Summary:**

Red foxes (*Vulpes vulpes*) are largely present in Italian wooded areas and often reach urban environments. These animals are susceptible to several bacterial and protozoan pathogens that are able to affect dogs and humans. Foxes may harbor arthropod-borne microorganisms, enteropathogens and leptospirae, and thus represent a potential source of infections for other animals. Previous surveys in fox populations have usually focused on few of these pathogens, whereas in the present investigation, the occurrence of several bacterial and protozoan pathogens have been investigated: *Salmonella* spp., *Yersinia* spp., *Listeria monocytogenes*, *Brucella* spp., *Ehrlichia canis*, *Anaplasma phagocytophilum*, *Coxiella burnetii*, *Leptospira* spp., *Neospora caninum*, *Hepatozoon canis*, *Babesia* spp. and microsporidia. Even though the survey was based on a small number of animals, the results suggested that red foxes in Central Italy are involved in the epidemiology of some infections.

**Abstract:**

Most surveys of pathogens in red foxes (*Vulpes vulpes*) have focused on particular agents. The aim of this study was to verify, with bacteriological and molecular analyses, the occurrence of the main bacterial and protozoan pathogens that are able to infect canids, in red foxes regularly hunted in Central Italy. Spleen, brain, kidney and fecal samples from red foxes were submitted to bacteriological and/or molecular analyses to detect *Salmonella* spp., *Yersinia* spp., *Listeria monocytogenes, Brucella* spp., *Ehrlichia canis, Anaplasma phagocytophilum, Coxiella burnetii, Leptospira* spp., *Neospora caninum, Hepatozoon canis*, *Babesia* spp. and microsporidia. Two (9.1%) strains of *Yersinia enterocolitica* biotype 1 and 2 (9.1%) of *Yersinia frederiksenii* were isolated from 22 fecal samples. Among the 22 spleen samples, seven (31.8%) were PCR-positive for *H. canis* and 3 (13.6%) for *Babesia vulpes*. Kidneys from two (2.9%) foxes, among 71 tested, were PCR-positive for *L. interrogans*. Even though the analyses were carried out on a small number of animals, the results suggested that red foxes from the selected geographic area may act as reservoirs of some investigated pathogens.

## 1. Introduction

Red foxes (Vulpes vulpes) are the most widespread wild carnivore in Italy. They are largely present in forest and lightly wooded areas that are typically found in agricultural landscapes. Foxes often reach urban and peri-urban environments in search of food, and therefore they can act as possible reservoirs for many pathogens that are able to infect domestic animals, mainly dogs, and humans [1]. In fact, foxes and dogs often are affected by the same pathogens, which can also be zoonotic. Bacterial enteropathogens, such as *Salmonella* spp. and *Yersinia enterocolitica*, can cause diseases in both dogs and humans, and reproductive disorders can be due to *Brucella* spp. and *Listeria monocytogenes* [2,3]. Furthermore, pathogenic *Leptospira* may affect dogs and people, causing severe, often lethal, disease [4].

Arthropod-borne bacteria and protozoa usually affecting dogs, such as *Ehrlichia canis, Anaplasma phagocytophilum*, *Hepatozoon canis*, *Babesia* sp. and *Encephalitozoon cuniculi* have been found in foxes in different European areas [1,5,6,7,8]. Conversely, dogs can act as spreaders of *Neospora caninum* oocysts into the wild, contributing to maintaining the life cycle of this parasite, which severely affects ruminant health [9].

Hunting dogs have a higher risk than pets of acquiring arthropod-borne pathogens due to increased frequency of tick exposure. Furthermore, they can come in contact with urine and/or feces of infected foxes that harbor pathogens in kidneys and/or the intestine tract.

Surveys about the spreading of bacteria and protozoa affecting canids in red foxes living in Italian areas are usually limited to some pathogens [1,10,11,12,13,14,15,16,17]. In view of the scanty data about the health status of the fox population in Italy, the aim of the present study was to verify, with bacteriological and molecular analyses, the occurrence of the main bacterial and protozoan pathogens, which can infect canids in red foxes regularly hunted in Central Italy.

## 2. Materials and Methods

### 2.1. Sampling

Twenty-two adult red foxes shot during the regular hunting seasons of 2016, 2019, and 2021 in the Province of Pisa (43° N, 10–11° E) were examined.

Foxes were brought by hunters to the Department of Veterinary Sciences, University of Pisa, within 48 h of being shot. They were then submitted to post-mortem examinations and samples from spleen, kidneys, brain, and intestine were collected. All samples were kept at 4 °C for max 24 h until bacteriological and molecular analyses were executed.

Feces present in the intestine were submitted to bacteriological analyses, whereas all tissue samples were used for the DNA extraction.

DNA from kidneys and brains previously (hunting season 2012–2015) collected from 49 adult red foxes for other purpose, and stored at –20 °C, were included in the present investigation.

### 2.2. Bacteriological Analyses

*Salmonella* spp.

Each fecal sample was submitted to procedures previously described [18]. Briefly, about 1 g of feces was incubated in 9 mL of buffered peptone water at 37 °C for 24 h. One mL of this culture was transferred into 10 mL of Selenite Cystine Broth (Oxoid Ltd., Basingstoke, UK) and 0.1 mL into 10 mL of Rappaport Vassiliadis Broth. The tubes were incubated at 37 °C for 24 h and at 42 °C for 24 h, respectively. One loopful from each broth culture was streaked onto Salmonella-Shigella Agar (Oxoid) and Brilliant Green Agar (Oxoid) plates. After incubation of the plates at 37 °C for 24 h, suspected colonies were submitted for biochemical characterization.

*Yersinia* spp.

After enrichment for each fecal sample in Peptone Sorbitol Bile Broth (Oxoid) for 21 days at 4 °C, a loop of the broth culture was sub-cultured onto Cefsulodin Irgasan Novobiocin (CIN) Agar (Oxoid) and the plates were incubated at 30 °C for 48 h. Suspected colonies were submitted to biochemical tests to determine the species [19] and confirmation was performed using the API20E biochemical gallery (bioMerieux, Marcy l’Etoile, France). *Yersinia enterocolitica* isolates were successively characterized on the basis of biochemical tests to distinguish the biotype [20].

*Listeria monocytogenes*

*Listeria* strains were isolated according to the ISO 11290 method with some modifications. Feces were introduced into 10 mL Half-Fraser broth (Oxoid) and incubated at 30 °C for 24 h. Subsequently, 0.5 ml of the primary enrichment cultures were transferred to 4.5 mL of Fraser broth and incubated at 37 °C for 48 h. A loopful of secondary enrichment was streaked onto Agar Listeria Ottaviani Agosti (ALOA) (Biolife, Milan, Italy) (Oxoid) and incubated at 37 °C for 24–48 h.

### 2.3. Molecular Analyses

DNA extraction was carried out on about 15 mg of each tissue specimen using Tissue Genomic DNA Extraction Kit (Fisher Molecular Biology, Trevose, PA, USA) and according to the manufacturer’s instructions. DNA samples were stored at 4 °C until used as a template in the PCR assays.

DNAs extracted from 22 spleens were employed in PCR tests to detect *E. canis, A. phagocytophilum, C. burnetii, Brucella* spp., *Babesia* sp., and *H. canis.* DNA samples obtained from 71 brains were used to search *N. caninum* and microsporidia; DNAs from 71 kidneys were used to detect microsporidia, *C. burnetii* and *Leptospira* spp.

PCR assays were performed following the protocols previously reported and using primers and conditions summarized in Table 1.

Sterile distilled water instead of DNA was used as negative control in each PCR assay. As positive controls, DNA extracted from commercial IFAT slides (Fuller Laboratories Fullerton, CA, USA) containing the following pathogens were employed: *A. phagocytophilum, E. canis, B. canis, C. burnetii, H. canis,* and *N. caninum.* DNA from the brain of rabbit affected by *E. cuniculi*, DNA from cultures of *L. interrogans* (serovar Hardjo) and *Brucella ovis* were added as positive controls as well.

All PCR assays were performed using the EconoTaqPLUS 2xMaster Mix (Lucigen Corporation, Middleton, Wisconsin, USA) and an automated thermal cycler (SimpliAmp™ Thermal Cycler, Applied Biosystems, Waltham, Massachusetts, U.S.A.). PCR products were analyses by electrophoresis on 1.5% agarose gel at 100 V for 45 min. Amplicons were sequenced by a commercial laboratory (BMR-Genomics, Padova, Italy). Sequences were assembled and corrected by visual analysis of the electropherogram using Bioedit v.7.2, then compared with those available in GenBank using the BLAST program (http://www.ncbi.nlm.nih.gov/BLAST, accessed on 15 September 2022).

## 3. Results

Bacteriological examinations carried out on the 22 tested fecal samples isolated four (18.2%) *Yersinia* strains and two (9.1%) *Y. enterocolitica* biotypes 1 and 2 (9.1%) *Y. frederiksenii.* No *Salmonella* or *L. monocytogenes* were isolated.

PCR assays on DNAs from spleens detected 3 (13.6%) animals positive for *Babesia vulpes* and 7 (31.8%) for *H. canis*. All 22 foxes spleens were negative for *E. canis, A. phagocytophilum, C. burnetii*, and *Brucella* spp.

DNAs from the 71 brain samples were negative for *N. caninum* and microsporidia.

PCR assays carried out on DNAs from the 71 kidneys resulted negative for microsporidia and *C. burnetii*, whereas three samples were positive for *Leptospira* genus; in detail, one (1.4%) sample was positive for non-pathogenic *Leptospira,* two (2.9%) for *L. interrogans,* as confirmed by the sequencing analysis.

## 4. Discussion

Even though the investigation was performed on a small number of red foxes, the results obtained by bacteriological and molecular analyses suggested that these animals do not act as relevant reservoirs of the investigated pathogens.

Few investigations about the occurrence of bacterial pathogens have been carried out in fox population in Italy, thus our data are not easily comparable.

Negative results for *Salmonella* disagree with a previous investigation that found a prevalence of 5.7% in red foxes living in Northern Italy [12], but they agree with a recent study that detected a 0% prevalence in *V. vulpes* in Northern Ireland [33]. However, the low number of samples investigated in our study could not reflect the real epidemiological situation and account for the discrepancy with the results of the other investigations.

Similarly, *L. monocytogenes* was found in 4.5% of *V. vulpes* in Poland [2], whereas no previous reports about the detection of this pathogens in Italian fox population are available.

The detection of *Y. enterocolitica* and *Y. frederiksenii* shows that these bacteria are circulating among wildlife in Italy, as well as other European countries, as also found by previous surveys in foxes and other wild mammals [2,17].

Foxes are susceptible to *Brucella* infection. *Brucella vulpis* and *B. microti* have been isolated from mandibular lymph nodes of *V. vulpes* in Austria [3,34]. In Italy, no cases of brucellosis in foxes have been reported, thus our results could really reflect the epidemiological scenarios in the investigated area, also considering that Tuscany is an officially brucellosis free region [35].

No foxes were found to be positive for *E. canis, A. phagocytophilum* and *C. burnetii*. These findings partially disagree with previous studies carried out in red foxes living in the same area that detected a different prevalences. In fact, 16.6% of red foxes tested in 2007–2008 were positive for *A. phagocytophilum*, but none of them were positive for *E. canis* [10]. Conversely, red foxes examined in 2014–2016 were positive with a prevalence of 44.44% for *E. canis*, 1.96% for *C. burnetii* and 0.65% for *A. phagocytophilum* [1].

Studies carried out in foxes from other European regions found variable prevalences in relation to the investigated pathogen, period, geographic area, density of tick population. In fact, prevalence from 0% to 52% were found for *E. canis* [5,13,36,37,38], from 0% to 8.2% for *A. pahgocytophilum* [5,36,39], from 0% to 17% for *C. burnetii* [13,38,40].

Members of the genus *Leptospira* are tightly coiled spirochetes that are rapidly killed on drying, heating and exposure to detergents or disinfectants, but they remain viable for several weeks in stagnant alkaline water or wet soil [41]. *Leptospira interrogans* species includes several serovars distributed worldwide in relation to geographic areas and animal species involved in the epidemiology. Wild mammals have been recognized as reservoirs for many serovars [42]. Serological investigations showed the exposure of red foxes to Leptospira, sometimes with remarkable seroprevalences: 33.8% in Croatia [43], 34% in Slovenia [44], 47% in Spain [45], 26.3% in Poland [46]. Conversely, data about the occurrence of pathogenic leptospirae in urine and/or kidneys from foxes are very scanty. Millan et al. [47] found one PCR-positive among nine investigated foxes in Spain, whereas Ayral et al. [48] found 6.1% of foxes PCR-positive for pathogenic *Leptospira* in France.

In Italy, the little information about leptospiral infections in foxes that come from national serological surveillance reported 22.7% prevalence in the period 2010–2011 [4].

Even though molecular analysis did not allow us to identify the serovar of the found leptospirae, our findings show the circulation of pathogenic leptospirae in the investigated area and the role of red foxes as possible carriers of the spirochetes.

Foxes carrying pathogenic leptospirae can act as maintenance hosts and contribute to the spreading of these bacteria to other species that share the environment.

Hunting dogs are at high risk of infection during their activity. Moreover, foxes often reach urban areas, thus they may be source of leptospirae for pet dogs, too. Serovars most frequently found in foxes in previous studies are Icterohaemorrhagie, Bratislava, Pomona that are well known to cause severe diseases in dogs.

*Babesia vulpes* DNA was recovered in 13.63%. *Babesia vulpes* (formerly *Theileria annae*) was recently renamed [49] and has been reported in canine babesiosis throughout the Europe [49,50,51]. It is responsible for asymptomatic infections in foxes (its reservoir host) while in dogs causes anemia, thrombocytopenia and renal damage [49,52]. *Hepatozoon canis* DNA was identified in 31.81% *V. vulpes*. The parasite is not a zoonotic agent, but can represent an important pathogen mostly for hunting dogs, more frequently exposed to ixodid ticks. Such data confirm the occurrence of these tick-borne parasites in the fox population examined, as previously reported [1].

Conversely, *N. caninum* DNA was not found in selected tissues. It is an apicomplexan protozoon responsible for reproductive disorders in ruminants and neuromuscular disease in dogs. The parasite has a cosmopolitan distribution and dog, wolf and coyote act as final hosts, shedding oocysts which sporulate in the environment. Oocysts are infective for several intermediate hosts such as cattle, where *N. caninum* can transmit transplacentally as well [53]. Carnivores can become infected by ingesting tissues of intermediate hosts. Foxes are considered as natural intermediate hosts for the protozoon, while fail to excrete oocysts [54] and rarely develop clinical signs [55]. Seroprevalences in Europe range from 0% [56,57] to 26.4% [58]. In surveys similar to the present study parasite DNA was not recovered from red foxes in Saxony [59] nor in Czech Republic [60], while was identified in 4.6% animals in a previous study in Czech Republic [61], in 4.8% from United Kingdom [62], in 9% to 18% from Slovakia [58], and 10% from Spain [54]. Our results are also supported by the lack of seropositivity in 253 foxes living in the same area (unpublished data), examined by IFAT in the last 10 years.

*Encephalitozoon cuniculi* was not reported from the examined brains. This is a microsporidian parasite associated with its natural host (*Oryctolagus cuniculus*) and has a broad range of hosts, with zoonotic potential, inducing neurologic, ocular and renal clinical signs. The parasite is responsible for clinical forms in blue and silver foxes [63]. Red foxes are reported to be infected in Ireland with about 0.7% prevalence [64], in Czech Republic with 3.8% [61], while in a further study in the same area microsporidian DNA was not detected [60].

## 5. Conclusions

Even though our study investigated a small number of red foxes, the obtained results showed that these animals can harbor bacteria and protozoans transmissible to dogs and humans. Foxes infected by *Yersinia* spp. seem to be involved in the epidemiology of these pathogens contributing to environmental contamination with their feces. Further studies are necessary to better understand the role of red foxes in the epidemiology of leptospirosis, because our survey detected *L. interrogans* DNA in kidneys, but it was not possible to verify if the animals had live leptospirae in their urine.

Furthermore, the survey suggested the role of red foxes as reservoirs of canine pathogens such as *B. vulpes* and *H. canis.*

## Figures and Tables

**Table 1:** PCR primers and conditions employed in the assays for the detection of each pathogen.

Pathogen	Amplicons (Target Gene)	Primers Sequence (5′–3′)	PCR Conditions	Reference
*Anaplasma* *phagocytophilum* *	932 bp (16 S rRNA) [First PCR]	GE3a (CACATGCAAGTCGAACGGATTATTC) GE10r (TTCCGTTAAGAAGGATCTAATCTCC)	95 °C–30 s 55 °C–30 s 72 °C–1 min	[21]
	546 bp (16S rRNA) [Second PCR]	GE9f (AACGGATTATTCTTTATAGCTTGCT) GE2 (GGCAGTATTAAAAGCAGCTCCAGG)		
*Babesia*	560 pb (ssrRNA)	Mic1 (GTCTTGTAATTGGAATGATGG) Mic2 (CCAAAGACTTTGATTTCTCTC)	95 °C–30 s 50 °C–30 s 72 °C–1 min	[22]
*Brucella* spp.	905 bp (16SrRNA)	F4 (TCGAGCGCCCGCAAGGGG) R2 (AACCATAGTGTCTCCACTAA)	95 °C–30 s 54 °C–1 min 72 °C–1 min	[23]
*Coxiella burnetii*	687 bp (IS1111a)	Trans-1 (TATGTATCCACCGTAGCCAGT) Trans-2 (CCCAACAACACCTCCTTATTC)	95 °C–30 s 64 °C–1 min 72 °C–1 min	[24]
*Ehrlichia canis* *	478 bp (16S rRNA) [First PCR]	ECCf (AGAACGAACGCTGGCGGCAAGC) ECBr (CGTATTACCGCGGCTGCTGGCA)	95 °C–1 min 55 °C–2 min 72 °C–1.5 min	[25,26]
	389 bp (16S rRNA) [Second PCR]	ECA (CAATTATTTATAGCCTCTGGCTATAGGA) HE3r (TATAGGTACCGTCATTATCTTCCCTAT)		
*Hepatozoon* spp.	660 pb (18S rRNA)	HepF (ATACATGAGCAAAATCTCAAC) HepR (CTTATTATTCCATGCTGCAG)	95 °C–30 s 57 °C–30 s 72 °C–1 min	[27]
*Leptospira* spp.	103 bp (16S rRNA) [*Leptospira* genus]	16S-P1 (TAGTGAACGGGATTAGATAC) 16S-P2 (GGTCTACTTAATCCGTTAGG)	95 °C–30 s 60 °C–30 s 72 °C–1 min	[28]
	242 bp (*lipL32*) [pathogenic leptospirae]	LipL32–45F (AAGCATTACCGCTTGTGGTG) LipL32–286R (GAACTCCCATTTCAGCGA TT)	95 °C–30 s 58 °C–30 s 72 °C–1 min	[29]
	450 (*rrs2*) [for sequencing]	rrs2F (CATGCAAGTCAAGCGGAGTA) rrs2R (AGTTGAGCCCGCAGTTTTC)	95 °C–30 s 58 °C–30 s 72 °C–1 min	[30]
*Neospora caninum*	337 pb (Region NC5)	Np6 + (TCGCCAGTCAACCTACGTCTTCT) Np21 (CCCAGTGCGTCCAATCCTGTAAC)	95 °C–30 s 63 °C–30 s 72 °C–30 s	[31]
Microsporidia	250–280 pb (18S rRNA)	V1 (CACCAGGTTGATTCTGCCTGAC) PMP2 (CCTCTCCGGAACCAAACCCTG)	94 °C–30 s 60 °C–30 s 72 °C–30 s	[32]

Legend. * Nested PCR.

## Data Availability

All data are reported in the manuscript.
